# Ethnobotany of *Heracleum persicum* Desf. ex Fisch., an invasive species in Norway, or how plant names, uses, and other traditions evolve

**DOI:** 10.1186/1746-4269-9-42

**Published:** 2013-06-24

**Authors:** Torbjørn Alm

**Affiliations:** 1 Torbjørn Alm, Tromsø museum, University of Tromsø, N-9037, Tromsø, Norway

**Keywords:** Giant hogweed, Heracleum persicum, Heracleum mantegazzianum, Vernacular names, Children’s games, Pest management

## Abstract

**Background:**

*Heracleum persicum* was introduced to Norway as an ornamental in the 1830′s. Towards the end of the 19th century, it started spreading outside gardens, later to become a frequent sight in the major towns and settlements of North Norway – and a veritable pest plant. During the last 100 years or so, a substantial ethnobotanical tradition related to the species has evolved, demonstrating that folk knowledge is not only forgotten and lost, but also charting new terrain.

**Methods:**

This survey is based on data extracted from all relevant publications, including botanical literature, travel accounts, newspaper notes, etc., as far as they have come to my attention. In addition, information on vernacular names and various uses of the *H*. *persicum* in Norway has been extracted from my own, substantial archive of interviews, questionnaires, and correspondence related to the ethnobotany of Norway.

**Results:**

Where extant, *H*. *persicum* tends to be known to everyone, even by city dwellers who otherwise generally neglect plants. People tend to love or hate it, and in Tromsø, the largest town of northern Norway, the species has become more or less emblematic of the city. Both here and in other areas of northern Norway, it is referred to by a variety of vernacular names, partly borrowed from other species, partly derived from the Latin genus name, and partly coined for this species only. In the latter group, *tromsøpalme* (‘the palm of Tromsø’) has proved by far the most popular invention. It was seemingly first used (and coined) by German soldiers during the World War II occupation of Norway, but now largely replaces other vernacular names. The plant is still popular with children, who frequently play in and with it, whereas adults have been more prone to speculate on its origins – and how to get rid of it. Salt is the most popular “herbicide” for this purpose.

**Conclusions:**

Over the years, *H*. *persicum* has accumulated at least twenty different vernacular names in Norway, and a variety of other traditions. By necessity, all these traditions are less than 180 years old, showing that even modern and urban societies may produce a substantial body of plant lore, which certainly merits ethnobotanical attention.

## Introduction

Ethnobiologists lament the loss of traditional knowledge across cultures worldwide, and frequently express their intention to salvage what is still left (e.g., [1-3]). “Westernization”, or perhaps rather the influence of the modern world, is frequently blamed [4,5]. Locally, over-exploitation and eradication of relevant species may potentiate the loss of local knowledge [6]. In some cases, it is even claimed that there is little or nothing left to collect, as suggested e.g. in a comment on the use of wild plants in pre-industrial Scandinavia: “Most of this knowledge is now forgotten and opportunities to gather data in the field are dwindling” ([7]: 153), thus leaving only archival and published records as extant sources. In their review of ethnobiological research in Europe, Pieroni and others noted more generally that “Fieldwork is still possible, especially in eastern and southern Europe” ([8]: 205). Several recent works, e.g. the large compilation of British plant lore provided by Roy Vickery [9], a recent study of old and new food plants in Europe [10], and my own experience from Norway, would rather suggest that field work is both possible and fruitful in many north European countries as well. It is obviously true that vernacular plant names and plant uses related to traditional subsistence farming are rapidly disappearing. Despite this, selected elements of the old folk plant knowledge may live on, simply because they are still considered useful and relevant, e.g. when traditional medicinal plants provide better cures than doctors may offer – as noted in Norway e.g. for treating herpes zoster with *Linnaea borealis* L. [11], and colds and rheumatism with a decoction of *Rhododendron tomentosum* (Stokes) Harmaja [12].

Restricting the scope of ethnobotany to traditional lifestyles and the past presents a strictly limited view of what the field should cover. In some ways, it is rather similar to the (mainly) American notion, as expressed e.g. by Richards Evan Schultes when visiting Britain, “The British Isles have no ethnobotany” (see [9]: vii), i.e., that only traditional knowledge of indigenous peoples has any claim to being ethnobotany; a limitation only conceivable in a nation (U.S.A.) consisting mostly of immigrants. Still, it ignores the fact that any immigrant group will bring portions of its own plant knowledge and traditions, often to some extent adjusting it to the flora of their new homeland, and thus merging old and new into novel bodies of ethnobotanical knowledge, as has been well documented e.g. in Europe [13,14].

Anyone, no matter their ethnicity, has some relationship with plants and animals, which is bound to produce at least some knowledge or tradition worthy of ethnobiological interest. Modern city and town dwellers may have much less contact with nature than their ancestors, but they are still bound to meet, eat, like and dislike a substantial number of plants. In doing so, they will obviously use a number of vernacular names, coining new ones as needed, and frequently accumulating a variety of other lore in the process.

This paper provides an example of such modern ethnobotany, by compiling vernacular names and plant lore, including uses, related to *Heracleum persicum* Desf. ex Fisch. in Norway. It was introduced as an ornamental to the far north of the country in 1836 [15]. Thus, the entire body of tradition presented here has accumulated in less than 180 years – and most of it probably after 1900, when the species started spreading outside gardens in earnest. It was soon to become a veritable pest plant, and is now duly black-listed in Norway as an aggressive alien [16].

## Material

The present study is based on a variety of material, extracted partly from my database of publications providing data on plant names and uses in Norway (currently running into some 7000 references), data found in a few archival sources; and, in particular, records in my own extensive collection of ethnobotanical data collected through interviews, questionnaires, and correspondence, over the last 35 years or so. These latter are referred to by year and record number, e.g. EBATA 1978:23 (see Additional file 1). Having spent most of my life in areas with abundant *Heracleum persicum*, I have frequently run into people who possessed various kinds of local knowledge related to the species.

### History

Large *Heracleum species* were fashionable in 19th century European gardens, not least due to their stately growth. Fruits of several taxa were imported and sown, but by far the most important species was *H*. *mantegazzianum* Sommier & Levier from the Caucasus [17] – now an invasive pest plant in many countries (e.g. [18-24]), and subject to numerous studies on control measures (e.g. [25-32]). In Norway, *H*. *mantegazzianum* is largely confined to the country’s southernmost parts [33-35].

Further north, in the coastal areas of central and northern Norway, another large, escaped *Heracleum* is a familiar sight, and may predominate in towns, ruderal areas, abandoned fields and along sea-shores [15,34,36-38]. The species found here differs from *H*. *mantegazzianum* in a number of characteristics [35,39]. Compared to *H*. *mantegazzianum*, the “northern” taxon is a smaller plant, rarely exceeding 2.5 m, and a true perennial, flowering repeatedly from the same root, whereas *H*. *mantegazzianum* is monocarpic, and dies after flowering.

For a long time, the identity of the plant found in the north of Norway remained a mystery, and various provisional names have been used for it, including *H*. *panaces*, *H*. *giganteum*, and, in 20th century Scandinavian literature, *H*. *laciniatum*. Recently, a DNA study of large *Heracleum* species in Europe [20] provided genetic evidence that the North Norwegian plant belongs to *H*. *persicum* – an identification previously suggested, but ultimately rejected by [40], who found it did not match herbarium specimens from Turkey, differing e.g. in the shape and length of fruit vittae [41]. Thus, it could possibly belong to an as yet unidentified hybrid, though for the time being, *H*. *persicum* is the most appropriate name [35], and the one that will be used here.

*H*. *persicum* remains the only large *Heracleum* species with a wide distribution in northern Norway, though the hybrid with the much smaller *H*. *sphondylium* L. ssp. *sibiricum* (L.) Simonk., first reported by [42], is now becoming common in some town areas [43,44]. *H*. *mantegazzianum* has only been reported from a couple of stations in Tromsø [35,44-46], and does not seem to thrive in the north.

The nomenclatural problem is beyond the scope of this paper, and of little bearing in our context. What is needed here, is an outline of the species’ history in the north, as far as this is known [15,38]. The first mention of a large *Heracleum* species in northern Norway is found in the travelogue of W. Christy, a Briton who visited northernmost Norway in 1836 [47]. He brought with him fruits of a “stately” *Heracleum*, which he distributed at some places in western Finnmark, e.g. at Kåfjord in Alta. They were also used to “embellish” a graveyard in Hammerfest [47,48]. Soon after, large *Heracleum*s featured in the gardens of the Alta area. From there, plants were brought to Tromsø and a number of other localities in northern Norway.

In his account of a botanical expedition to northernmost Norway in 1864, Thore M. Fries noted that a large *Heracleum* species was a favourite item of the small gardens along the coast of Finnmark; he saw it e.g. at Gjesvær, close to the North Cape ([49]: 30). Later travellers in north Norway also mentioned the plant, e.g. von Heuglin ([50]: 39) and Escard ([51]: 20), who both saw it in Tromsø (Troms). Philip Sewell, who visited northern Norway in 1888, noted that “some coarse species of *Umbelliferæ*” – obviously *Heracleum persicum* – was cultivated in the area ([52]: 458). In his review of useful plants in Norway, including ornamentals, F. C. Schübeler noted that what he called *H*. *panaces* was grown at many places in northern Norway, all the way northwards to Gjesvær (71°7′ N), that plants were attaining 2.5 m in height, and producing ripe fruits even at Vardø (70°22′ N), with a July mean temperature of less than 10°C, i.e. within the low arctic ([53]: 283, [54]: 232–233). At Hammerfest, cultivation was recorded again in 1894 ([55]: 88), and in the Sør-Varanger area of easternmost Finnmark, it was noted in 1895 that a large *Heracleum* species had been introduced as an ornamental about thirty years ago, i.e. in the 1860′s, and was now growing “almost as a weed at inhabited places” ([56]: 89).

Despite being frequently mentioned from 19th century Finnmark, the species thrives much better along the coast of Nordland and Troms, not least in Tromsø. It was introduced here about 1850, with plants brought from Alta [38,57-59]. Photographs from late 19th century Tromsø frequently show large *Heracleum* stands embellishing gardens, and the species is mentioned in some 19th century accounts of the town and its people (e.g. [60]). Towards the end of the century, the plant was spreading outside gardens, and in his flora of Tromsø, published in 1901, Andreas Notø considered the species an established member of the local flora ([61]: 133). Thus, along with Reusch [56], Notø is the first to suggest that *H*. *persicum* had expanded beyond cultivation, and was getting established as an anthropochore.

Unfortunately, late 19th and early 20th century botanists took little notice of introduced plants, generally neglecting them, and thus depriving us of data that would make it possible to follow the subsequent spread in some detail. By the early 20th century, *H*. *persicum* formed large stands outside gardens at least in Harstad [62] and Tromsø in Troms, and possibly at Honningsvåg in Nordkapp, Finnmark ([63]: 65). From the 1940′s onwards, the species was included in the major Norwegian floras. Whereas Rolf Nordhagen ([64]: 466) merely added a note that it was “cultivated all over North Norway”, Johannes Lid ([65]: 412) gave the species full listing, as “introduced in Tromsø and Tromsøysund”; revised in later editions to “introduced in Tromsø and elsewhere in north Norway” ([66]: 192), and “escaped, mostly in Troms and Finnmark” ([67]: 529).

## Results

### Origin according to folk tradition

The comment in [47] is the only concrete evidence of a crucial step in the history of *H*. *persicum* in Norway, namely its introduction through fruits – in this case imported via England. As noted by [48], *Heracleum* “seeds” were heavily advertised and marketed in Britain. Most buyers probably received *H*. *mantegazzianum*, but at least some batches of deviant derivation must have occurred, comprising *H*. *persicum* and perhaps other species as well. In 19th century Norway, a substantial part of the trade along the coast was with Germany, and it is certainly possible that *Heracleum* fruits were also imported from German sources. However, bearing in mind that the *Heracleum* species found in the northern parts of Norway deviates from the plants (mainly *H*. *mantegazzianum*) predominant elsewhere in western Europe, it is more likely that they derive from a single, deviant source, with the fruits brought by W. Christy as an obvious candidate.

Despite this, folk tradition in northern Norway frequently claims (or assumes) that the *Heracleum* plants had been brought from northern Russia. This area was also subject to lively trade connections in the 19th century, the so-called pomor trade (from a Russian word meaning “those that live at the coast”, i.e., Russians at the shores of the White Sea, including the major port of Archangelsk). A woman from Loppa in western Finnmark noted that “(…) my mother believed it came through the pomor trade” – i.e., from Russia (EBATA 2006:132). In Sør-Varanger, eastern Finnmark, a woman “had heard that someone in Alta got it from Russian merchants or [some similar source]” (EBATA 2009:27). Literature sources sometimes make the same assumption: “It was supposedly imported from somewhere in northern Russia” ([57]: 448) – but there is no evidence to support this. On botanical grounds, it is unlikely. There are several introduced *Heracleum* taxa on the Kola peninsula and in the adjacent White Sea area, but these belong to other species (including *H*. *mantegazzianum* and *H*. *sosnowskiy* Manden and a plethora of hybrids). Their introduction is comparatively recent, and mostly related to the experiments with growing plants transferred from other parts of Russia, carried out in the botanical garden at Kirovsk from the 1930′s onwards [68]. Less frequently, other origins are suggested. At Loppa in Finnmark, people claimed that the plant had been imported from Germany ([38]: 62).

### Vernacular names

So far, about twenty vernacular names have been recorded for *H*. *persicum* in Norway, mostly through my own collections (Table 1). The names fall into four different groups, providing an interesting insight in how people find names for a new species:

(1) ***Derivations from the Latin name***. As a garden plant, *H*. *persicum* may have been distributed as packets of “seeds” labelled simply *Heracleum*, i.e. with the genus name only. The Latin name was certainly known to many of the early cultivators. It was soon borrowed and adopted as a Norwegian name, in slightly modified form: *herakleum*. No other vernacular name seems to have been used in the late 19th and early 20th century. It was used e.g. at Gressholmen in Harstad about 1900: “We called it *herakleum* only.” (EBATA 1978:23). It remained the only name used in the area until about 1950, as elsewhere in Troms (see below).

With little knowledge of Greek mythology among the layman, *herakleum* was simply a somewhat foreign label. People did not hesitate in changing it. So far, this has proved the most productive source of vernacular names. Further derivations fall in two subgroups:

(a) Abbreviations that simply make the name shorter, but add no meaning: This group has been particularly productive, resulting in a whole series of vernacular names (*arakla*, *araklia*, *auraklum*, *herakla*, *rakelung*, *rakleum*, *uraklium*), with further examples (*høyrakel*, *orakleum*, *ørneklo*) in the next subgroup.

(a) Re-interpretations, which make an attempt at inserting meaning into the name, by changing at least parts of it into well-known Norwegian terms: *Høyrakel* is a typical example: *høy* may mean both ‘high’ and ‘hay’, wheras *rakel* means nothing at all, but bear at least some superficial resemblance to words like *rake*, ‘rake’, and *rekel*, ‘long being’ or ‘long person’. Among the vernacular names used in Tromsø, *orakleum* carries at least a semblance to *orakel*, ‘oracle’, though there is nothing to suggest that any fortune-telling use was invented or attempted.

In a single case, at Andøya in Nordland, a name in this subgroup is likely to derive from a number of local names (*arakla*, *araklia* etc.) in the former subgroup, reinterpreted as *ørneklo* ‘eagle claw’. It was used in the village of Bleik: “When I grew up at Bleik (I was born in 1949), nobody used any other name than *ørneklo*.” (EBATA 2005:40).

(2) ***Names borrowed from other taxa***. Folk tradition frequently re-uses existing names, in particular if a species is missing in an area, so that its name is “available” – transposition in the terminology of Grenand ([69], see also [70]). For instance, *blåveis*, the predominant name for *Hepatica nobilis* Schreb. in Norway, is frequently used in the northernmost part of the country, where the species is absent. Here, the name is usually transferred to *Geranium sylvaticum* L. In similar fashion, *liljekonvall*, the “southern” name for lily-of-the-valley *Convallaria majalis* L., is used for *Pyrola* spp. in northern Norway, where the former is missing. In both cases, similarities are restricted to the flower colour.

In the case of *Heracleum persicum*, vernacular names have been borrowed from other large umbellifers, which at least look slightly similar, mainly *Angelica archangelica* L., e.g. *sløyke* in Hadsel, Nordland, and Tromsø, Troms [15], and *sløke* in Nordkapp, Finnmark. With some dialectal variations, *sløke* is the most frequent Norwegian name of *A*. *archangelica* in northern Norway ([71]: 217), but as noted at Nordkapp, it could also be used for *Heracleum:* “They called it *sløke*. That was the name they used.” (EBATA 2006:101). People were certainly aware that *H*. *persicum* was a different plant, e.g. at Hadsel in Nordland: “They called it *sløyke*. Up in the mountains, there is also real *sløyke*, the one that you can eat.” (EBATA 1988:13). Further south in Nordland, at Sømna in Helgeland, people have used *lur* as a name for *H*. *persicum*. It is a more general term, deriving from the Norse *luðr*, denoting a hollow object [72] – but frequently used for large umbellifers due to their hollow stems.

(3) ***Inventions***. The third major group of vernacular names for *H*. *persicum* has no parallel in other species. They are inventions, coined solely as labels for this introduced species – or neologies in the terminology of Grenand [69]. *Stormtræ* ‘storm tree’, recorded in the wind-swept outer-coast town of Andenes in Andøy, Nordland, was motivated by the resilience of the dry stems; they were the only herbs able to withstand the strong winter gales (EBATA 1984:11). Three names incorporating the term *rotte*- ‘rat’, e.g. *rottefrø* ‘rat seed’, are obviously pejorative.

**Table 1:** **Norwegian vernacular names for *Heracleum persicum***

**Name**	**Area and source**
***a) Vernacular names derived from the Latin genus name***	
Arakla	Nordland: Andøy (EBATA 2005:46)
Araklia	Nordland: Andøy (EBATA 2005:47)
Auraklum	Troms: Tromsø (EBATA 2007:24)
Herakla	Nordland: Andøy (EBATA 2005:38)
Herakleum	Troms: Harstad (EBATA 1978:22, 1978:23), Lenvik (EBATA 2004:23, 2007:88), Tromsø (EBATA 2005:32, 2005:34, 2006:133, 2013:3)
Heraklium	Troms: Tromsø (EBATA 2005:32)
Høyrakel	Troms: Karlsøy (EBATA 1995:1)
Orakleum	Troms: Tromsø (EBATA 2005:19, 2005:21)
Rakelung	Troms: Skjervøy ([71]: 384)
Rakleum	Troms: Harstad ([71]: 384)
Uraklium	Finnmark: Loppa (EBATA 2006:132)
Ørneklo	Nordland: Andøy (EBATA 2005:40)
***b) Vernacular names borrowed from other species***	
Lur	Nordland: Sømna ([74]: 111)
Sløke	Finnmark: Nordkapp (EBATA 2006:101)
Sløyke	Nordland: Hadsel (EBATA 1988:13); Troms: Tromsø ([89]: 18)
***c) Innovations***	
Rottefrø	Troms: Tromsø ([71]: 384)
Rottegift	Troms: Harstad ([71]: 384); (EBATA 2007:94)
Rottegras	Troms: Kvæfjord (Hallfrid Christiansen, archival note at the University of Tromsø, dated 1948)
Stormtræ	Nordland: Andøy (EBATA 1984:11)
Tromsøplanten	Finnmark: Vadsø (EBATA 2003:14)
Tromsøpalme	Nordland: Andøy (EBATA 2005:39, 2005:46, 2005:51), Hadsel (EBATA 2006:69); Troms: Bjarkøy (EBATA 2010:32), Harstad (EBATA 1977:2, 1978:22, 1978:23, 2008:18); Skjervøy (EBATA 2005:44, 2006:1), Torsken (EBATA 2005:17), Tromsø (EBATA 2005:21, 2005:32, 2005:43, 2005:49, 2006:40, 2006:133, 2013:3); Finnmark: Alta (EBATA 2007:45), Båtsfjord (EBATA 1988:12), Hammerfest (EBATA 1999:5), Måsøy (EBATA 1994:3, 2006:88), Nordkapp (EBATA 2005:34, 2006:100), Vadsø (EBATA 2006:117, 2006:119)

By far the most successful invention is *tromsøpalme*, “the palm of Tromsø”, by now the most commonly used name for the species in Norway. It has also been chosen as the official Norwegian name. As such, it was introduced in the third edition of the Norwegian standard flora ([67]: 529). A rather simple derivation of the name was suggested by Ove Arbo Høeg ([71]: 384): “due to its massive occurrences at Tromsø, and its gigantic size, the plant is often called *tromsøpalme*.” Still, the origin of the name is somewhat obscure. The first mention I have been able to trace is in a German book on the flora and fauna of Norway, intended as reading for the German troops occupying Norway during World War II. According to the text, the plants “was called *Tromsö*-*Palme* by the soldiers” ([73]: 32). Thus, it its likely that the name was a German invention, probably intended as a pun, e.g. to contrast the conditions facing soldiers serving in the high north to those stationed in the Mediterranean area, and even more so in the African detachments. The linguist Hallfrid Christiansen was aware that the name had a somewhat jocular quality, commenting that the plant “was popularly and a bit jokingly called “Tromsøpalmen” ([74]: 111).

None of those I have questioned about *H*. *persicum* had any recollection of the name *tromsøpalme* being used earlier than in first post-war years. At Andøya in Nordland, the term was known shortly after World War II, perhaps slightly prior to 1949 (EBATA 2005:39). A similar dating was suggested in nearby southern Troms: “I cannot remember this name being used in the Harstad area before the war. I first became aware of it about 1950, I suppose.” (EBATA 2008:18). In similar fashion, *tromsøpalme* has supplanted an earlier vernacular name in Nordkapp, Finnmark: “They called it *sløke*. That was the name they used, what they said. *Tromsøpalme*, it was introduced just some years ago.” (EBATA 2006:101)

Even in Tromsø, other names previously predominated [75,76]: “We said *herakleum*, we did not say *tromsøpalme*. I would guess in 1947, 1948, we [still] used *herakleum*. And all of us said *herakleum*, we had not even heard of *tromsøpalme*.” (EBATA 2005:32). “We said *herakleum* when I was a child.” (…) “At least until after the war. Then, the *tromsøpalme* name became common.” (EBATA 2006:133). Some believed the latter name had gained support through the newspapers (EBATA 2005:19), which may well be correct.

As a vernacular name, *tromsøpalme* has been so successful that it is now largely supplanting all other vernacular names. Some of those I have interviewed could only remember that the plant previously went by some other, now forgotten name (e.g., EBATA 2010:32).

### Cultivation

**Figure 1:**
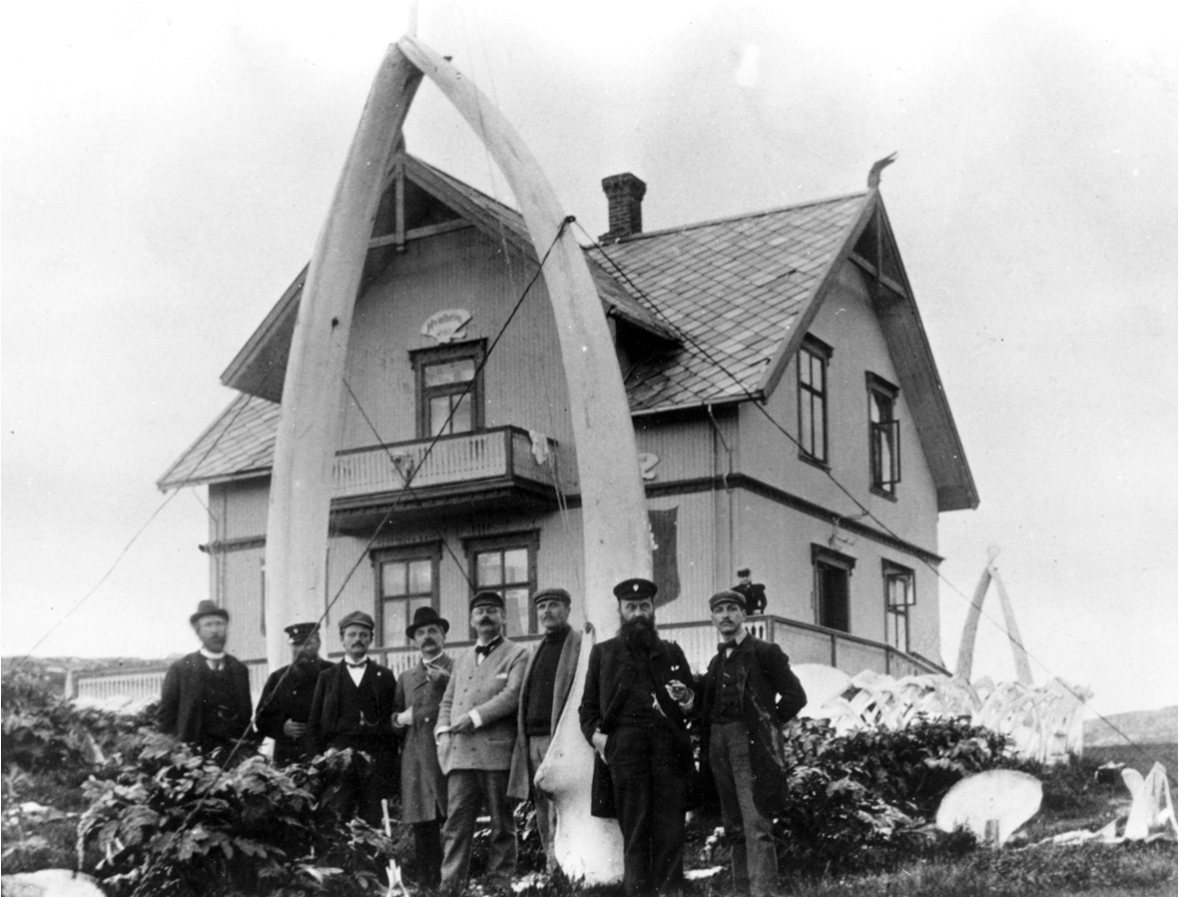
**An example of 19th century cultivation: Rows of *Heracleum persicum *surrounding a whaling station at the outer coast.** Skorøya in Karlsøy, Troms, 1898 (Photo archive of Tromsø museum).

Like *Heracleum mantegazzianum*, *H*. *persicum* was introduced to Europe as an ornamental. Old photographs from coastal North Norway frequently show large stands adorning gardens and summer houses (Figure 1), not least in Tromsø (for further examples, see [15]). As noted in the introduction, foreign visitors were frequently impressed by its vivid growth in the high north. This aspect is mentioned in a number of travel accounts, e.g. by François Escard, a French author, who visited Tromsø in 1884. Like everyone else, he failed to identify it correctly: “I would have liked to send some living specimens [to Bonaparte’s collection in France] of this stout *Heraclea sibirica*, which emits such a fine scent from all public and private gardens in Tromsø” ([51]: 20). Cultivation is also mentioned in an anonymous description of life at a parsonage at “70 degrees north”, i.e. somewhere in Troms or Finnmark: “Even the *Heracleum*s, which are here almost too much of a blessing, entice our eyes at this time [of year], for you can almost see that they are growing.” ([77]: 100).

Interviews provide some further glimpses of its use in gardens, e.g. at Dverberg in Andøy, Nordland: “According to what people said, *tromsøpalmen* was planted around the houses, as an ornamental and to provide shelter. As there is such a lot growing at the parsonage, it is assumed that the vicar’s family has planted *herakla* in the garden, which was established around 1870.” (EBATA 2005:38).

*Heracleum persicum* was still popular, and being introduced to new gardens, about 1900, e.g. at Reinøya in Karlsøy, Troms: “My grandma (…) had it in a garden there, at Reinøya. I believe it was introduced from Tromsø about 1900.” (EBATA 1995:1). A similar date was suggested at Honningsvåg in Nordkapp (Finnmark): “We’ve got it in the garden here, and it is about a hundred years old” (EBATA 2006:100). As is often the case on the outer coast, it was used to provide shelter: “There are perhaps twenty or thirty meters of it, as a sheltering wall” (EBATA 2006:100). It was put to similar use at Andøy in Nordland: “*Araklia* was used to provide shelter” (EBATA 2005:47).

Nowadays, cultivation is much less frequent, and primarily seen in some coastal villages of the high north, where trees and shrubs fail to grow. Exceptions occur, and *H*. *persicum* has been planted as an ornamental at some graveyards in comparatively recent times, e.g. at Laukvik in Lenvik, Troms (EBATA 2007:88).

### Decoration

The stout, dry stems and umbels of *Heracleum persicum* remain a favourite motive among photographers in Tromsø, especially in late autumn and winter. Such pictures are featured by the local newspapers every winter. The decorative, dried-out umbels have also frequently been brought indoors, e.g. in Målselv, Troms: “In the seventies, my aunt had dried flowering stems” – as a decoration. She kept them for years, but in the end, “they were thrown in the sea” (EBATA 2007:50). The umbels have also been used as topics for jewellery and similar small, decorative objects.

Several artists have found that the stems provide useful raw material. The collections at Nordnorsk kunstmuseum (Art museum of North Norway) in Tromsø include a large sculpture called *Log* by Stuart Frost, which is made entirely of rings cut from the hollow stem [78-80] – and he is not the only artist to have been inspired by it [81].

### Children’s games

*Heracleum persicum* is so large, and such a conspicuous feature of the flora, that it can hardly be overlooked. Still, a major cause of its ethnobotanical success, in terms of accumulating folk tradition, is obviously its frequent use in children’s games. The large, stout and hollow stems are eminently suitable for a number of purposes, not least as blowguns. Both dry and fresh stems could be used, e.g. in Harstad (Troms): “As children, we made blowguns from the stems. I remember a mild, liqorice-like taste, though we never chewed the stems.” (EBATA 2011:5). Such use involved a potential risk of sores, as noted at Brønnøysund in Nordland: «As children, we cut tubes of the stem, and used them for blowing rowan berries. Some got sores around the mouth, others not.” [82]. Similar games were played in Tromsø: “I remember Leif told me, they made blowguns from it.” (EBATA 2006:25). Here as well, some had recollections of a definite downside to this practice: “Don’t you get sore on your lips if you use them for blowguns? [It is] an old experience.” (EBATA 2006:4); “We tried them as blowguns, but they made our mouths sore.” In the latter case, the stems were fresh: “I think we took them in summer, when they had grown up. But perhaps we only tried once.” (EBATA 2005:19); “But we played, we used them as blowguns. Our mouths became sore, but we did not think about it.” (EBATA 2005:32). Others took precautions to avoid sores: “Those blowguns I remember, they were not from the green stems, but taken when they were dry. We knew they were dangerous when green.” (EBATA 2006:40). Further north, in Nordkapp (Finnmark), only dry stems were used (EBATA 2007:44).

Peas were the ammunition of choice – if available, whether the family consented to such use or not, e.g. in Tromsø: “Yes, peas – but we could not afford to buy them.” (EBATA 2005:19). If not, fruits of rowan *Sorbus aucuparia* L. provided a useful substitute: “And we used rowan berries, or stolen peas.” (EBATA 2005:32). The children at Honningsvåg in Nordkapp (Finnmark) followed suit: “The rabble I can remember, it was those boys who went about with blowguns and peas.” (EBATA 2007:44).

An alternative use of the hollow stems was to spray water. To do so, they were filled with water, and a smaller stem, e.g. of *Rumex longifolius* DC, was inserted to force the water forward and out. The children at Loppa in Finnmark played in this way: “We also made syringes and had water wars (…)” (EBATA 2006:132) (see also [83]: 105,106).

The stems were frequently used more directly as weapons, to hit or fence with, e.g. at Andøy (Nordland): “We used to chop off suitably long stems, remove the top and leaves, in order to use them as swords or canes for fencing” (EBATA 2005:46); “we used *arklia* stems from the previous year to fence with” (EBATA 2005:47); “the children would run about with it in the autumn” (EBATA 2005:39).

In my own childhood in Harstad, Troms, only dry stems were used. As such, they were certainly harmless, and would easily splinter. Other, less considerate users, e.g. in Tromsø, preferred fresh stems: “No, we used them fresh, it provided them with a fine weight.” (EBATA 2005:32). The stout stems were easily available even in winter: “I remember we once used frozen *tromsøpalmer* as weapons.” ([84]: 16). If combat with other children proved boring, an alternative target was close at hand: “And then we made war on *auraklumen*”, ie., the *Heracleum* stands (EBATA 2007:24).

Despite these useful properties, the large and dense stands as such were perhaps the most valued aspect of *Heracleum persicum* among children, not least in coastal areas devoid of forest, and with little to offer in terms of shrubs. The dense foliage provided something that was often considered as “jungles” – a fine area for hiding, and for games in general, e,g. at Hadsel in Nordland: “In a couple of gardens [at the house] where we lived until 1968 there was a lof of *tromsøpalmer* along the road, almost like a hedge. It was a fine place for hiding when we played hide and seek.” (EBATA 2006:69). Similar games were played in nearby Andøy: “The children used to run about there in the autumn.” (EBATA 2005:39); “We pretended to be Indians, making huts in the *araklia* jungle” (EBATA 2005:47).

The jungles certainly feature lively in the childhood memories of many of those who have grown up in Tromsø, for they would “play Indians in the *Heracleum* forest” ([85]: 17); “we played Tarzan in swimming suits only [there], and built huts from it” [86] “We made huts, lived down there.” (EBATA 2006:26); “we made tunnels there” (EBATA 2013:2); “We were hiding there, ran between the plants, and collected dry stems which we used for blowguns, waved or hit with (EBATA 2013:3). “There we made huts from dry *tromsøpalme* stems.” (…) “There were lots of *tromsøpalme* here in the old days. A jungle.” (EBATA 2006:40). ”Above the city center, we had a veritable “forest” [of *H*. *persicum*] at hand. Here, Robin Hood roamed with his men, and Tarzan, the king of apes. In the cinema, we had seen Indians and cowboys ride across the prairie with the dust raising high behind them. Deprived of horses, even they [i.e., the children pretending to be riding horses in the wild west] would fit under the leaves.” (…) “And last, but not least, quite a few small outlaws have bided their time here, searched for by their family, while the storm abated.” [87].

Children in Finnmark utilized the *Heracleum* stands in much the same way, e.g. at the island of Loppa: “A small forest of *Heracleum* which grew at the outskirt of the parsonage’s field provided a fine place for hiding.” ([83]: 105). Similar games occurred at Honningsvåg in Nordkapp: “We grew up with *tromsøpalmen*. For us, it was a veritable forest; we have no [real] forest.” (…) “I remember our wars very well.” “We played inside [the stand], and would hide there.” (EBATA 2005:34).

The hollow stems could also serve as a kind of telescope – or rather, a telescope look-a-like, as noted in Lenvik, Troms: “I remember my grandfather told me that they made binoculars of *tromsøpalme*. And since its juice is somewhat caustic, they had [sore] raccoon-like rings around the eyes” (EBATA 2005:31). Others would hardly believe that the plants could be dangerous, commenting that the children in Nordkapp, Finnmark “had fought and ravaged and torn apart and hit each other with these [plants], and never suffered [any harm]” (EBATA 2006:100).

Florivory, the habit of eating flowers, is common among children worldwide [88]. *Heracleum persicum* was no exception. At least by children in Tromsø, the young umbels were considered edible: “Yes, we ate the bud.” (EBATA 2006:26). “We ate them. They tasted like cauliflower.” “And I have spoken with several others who ate them. We never got ill.” (EBATA 2005:32). “We even ate *rogna* [the roe, i.e., the flower] of one» [86]. “*Sløyke*? Yes, it is the flower buds. You just unwrap them and eat. They taste like cauliflower, and look like it as well.” [89]. Others refrained from eating them: “We never ate *tromsøpalma*” (EBATA 2006:40); or were told to keep clear: “We were not allowed to touch it” (EBATA 2007:25).

Children experimenting with sigarettes might also find the *Heracleum* stands useful – as a hide-out, and not least because the plant’s strong smell would mask any tell-tale evidence of forbidden use of tobacco: “And then we went into *auraklumen*, for afterwards it would not smell” (EBATA 2007:24).

### Utility purposes

Except as an ornamental (and decoration), *H*. *persicum* is hardly considered a very useful plant in Norway. In the 1930′s, experiments were carried out in Tromsø to explore its potential as a fodder plant [58]. The cows willingly ate it, but as the milk got a distinct taste of *Heracleum*, the project was abandoned.

In folk tradition, only a single record mentions its use for a utility purpose, to light a fire, at Andøy in Nordland: “I have not heard that *herakla* was used for other purposes, but the stout and dry stems were sometimes collected and used as “firewood” to get turf burning.” (EBATA 2005:38). In Tromsø, I have also seen the dry stems collected for midsummer’s eve bonfires [75].

The use of hollow *Heracleum* stems to make flutes may be considered a spin-off of folk tradition, where other Apiaceae species are frequently used [90]. Similar *Heracleum*-based instruments are still sufficiently novel to arouse media interest [91-95].

### Pest management

From the late 19th century onwards, *H*. *persicum* has expanded into a range of habitats outside gardens, including ruderal areas, abandoned meadows, seashores etc. Slowly, people have come to realize that the once popular ornamental is an invasive weed – and one that is difficult or impossible to control. As a consequence, they have invented a number of methods to decimate or eradicate it. Some have noted that heavy grazing might suffice, especially if it took place in early spring, e.g. at Andøy (Nordland): “The sheep enjoyed the green sprouts of the *herakla*, and kept the area almost barren. This put an end to the *herakla*, and after a few seasons, the weed was gone.” (EBATA 2005:38). Heavy grazing in early spring was equally successful at Reinøya in Karlsøy (Troms): “The sheep used to eat it in spring. And in fact, they kept it down. But then, we made a fence, and the plants were out of reach due to the enclosure. And then it expanded” (EBATA 1995:1).

Chopping off the flowering stems before the fruits ripen is obviously effective in terms of halting local expansion, and was practiced e.g. at Nordkapp and Vadsø (Finnmark). Doing so may have been inspired by governmental or other advice (EBATA 2006:100, 2006:117).

A popular remedy or “herbicide” along the coast was salt, easily available e.g. from the many fish-handling plants [96-103]. People at Andøy in Nordland preferred this method: “To put down *arakla*, they poured half-a-bucket, or a whole bucket, of salt among the stems. The salt penetrated towards the root, and death occurred little by little.” (EBATA 2005:46). Another informant in the same area combined salt with cutting down the plants in early spring, allegedly a successful procedure (EBATA 2005:39). Similar measures were used further north as well, e.g. at Lenvik (Troms): “We used to take salt from the cold storage plant, and sprinkle outside the garden fence. Then, they will die, they do not sprout” (EBATA 2005:17). This technique was known in Tromsø as well, but perhaps not too successful: “I cut the stem and filled it with salt. And still, next year it [the plant] was as fine as before” (EBATA 2005:43). Some would first dissolve the salt in water, and sometimes even heat it to boiling before applying it (EBATA 2006:88, 2009:30).

Others tried to combat *H*. *persicum* by digging it up – at least in one case with the help of local boys looking for earthworms as bait (EBATA 2005:46) – although some suggest that bait from such soils catch no fish [99]. Others would pour solar oil on the roots, a more dubious choice in terms of environmental impact – the latter technique recorded from Lenvik and Tromsø in Troms (EBATA 2009:30, 2010:25). Others report similar use of ammonium chloride [99,100], gasoline [101], or paraffin [102]. At Måsøy in Finnmark, even sour milk had been tried, but boiling salt water proved more effective (EBATA 2006:88).

The control measures invented locally show some similarity with those tried and tested in a number of scientific studies. In both cases, grazing and herbicides [26-30] are the prime choices. However, salt water may seem a safer choice than glyphosate [32] – and perhaps worth a study.

### An emblematic plant

**Figure 2:**
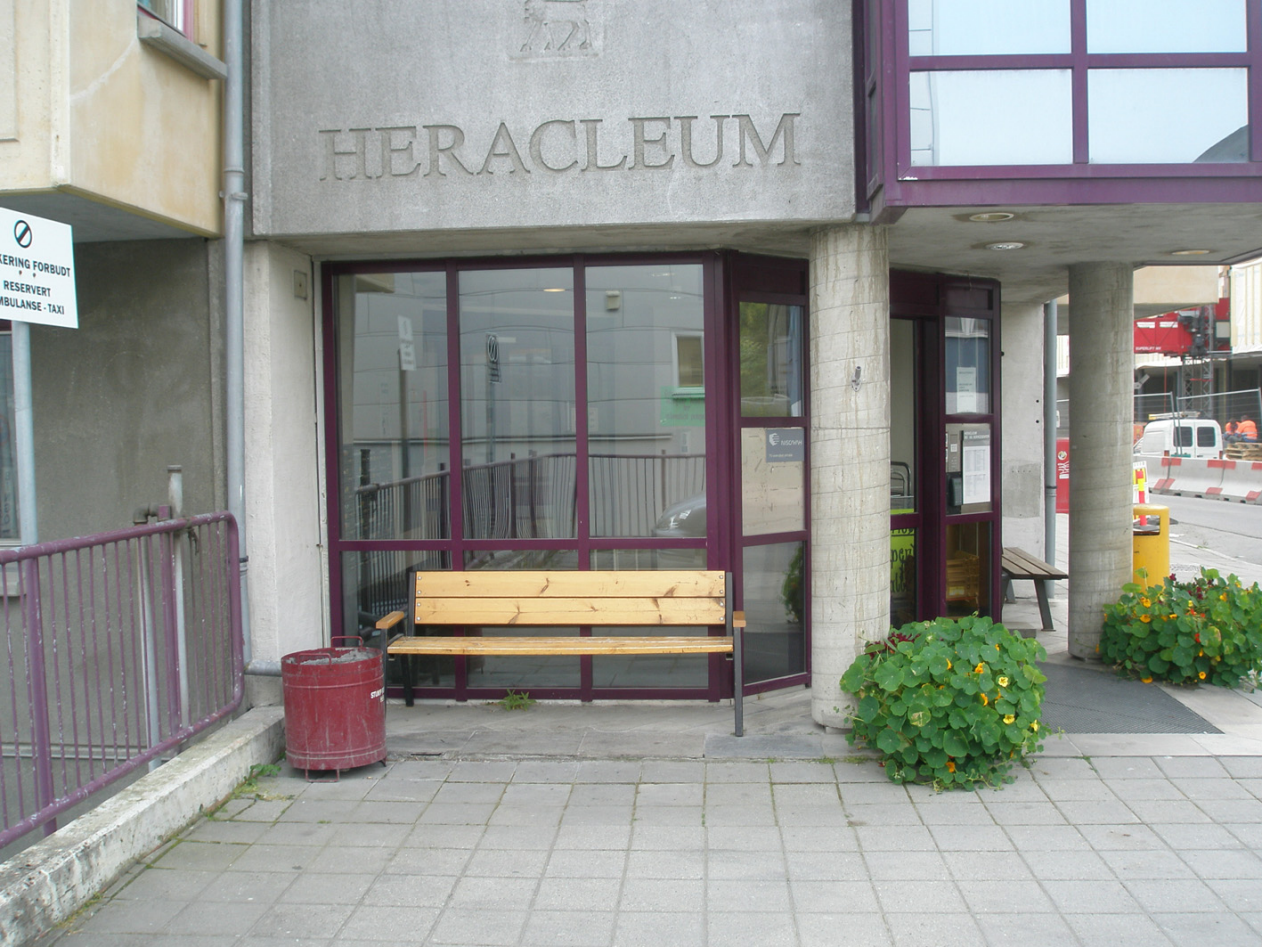
**Stimulated by the increasing predominance of *tromsøpalme *(‘palm of Tromsø’) as a vernacular name, *Heracleum persicum *has become emblematic of Tromsø, featuring in numerous contexts and giving its name e.g. to *Heracleum, *the city’s activity center for the elderly (August 29, 2012).**

In Tromsø, *H*. *persicum* has become more or less emblematic of the city. It is frequently photographed, and has been used in art and jewellery. The city’s activity center for the elderly (*Heracleum*) (Figure 2), an annual school revue running since 1990 (*Tromsøpalmen*), and one of the major prizes of the city’s annual international film festival (*Tromsøpalmen*) have all gained their names from it. *Heracleum persicum* or *tromsøpalmen* has become emblematic to such an extent that attempts at eradicating the species has met considerable opposition among locals. People are prone to defend the plant, contributing newspaper comments opposing any such action (e.g. [104-110]). Some claim that it forms part of the city’s “soul” or “identity” [107-112], advocating the formation of society of its friends, “*Palmens venner*” [113]. Others disclaim any harmful effect of the plant’s sap – usually by referring to its extensive use in children’s games (e.g., [86,114]). It has even been proposed that *H*. *persicum* cannot possibly be an introduced species, but rather an indigenous plant which may have survived the last ice age in caves (sic!) at Tromsøya [115]. Others have suggested replacing the reindeer now adoring the city emblem of Tromsø with *Heracleum persicum*[106,110,116-122].

Norwegian folk costumes (*bunad*) are popular garments for festive occasions throughout the country, despite being traditionally used only in some district. Areas with no traditional pattern have solved the problem by inventing new ones, and the one for Tromsø of course incorporates *Heracleum* umbels in its design. At the crown prince’s wedding in 2001, several suggested using a *tromsøpalme* as the city of Tromsø’s wedding gift, either a live plant specimen, or a glass engraving of it [123]. All this is solid evidence that *tromsøpalmen* is now deeply engraved in local lore and tradition.

## Discussion

With the earliest documented introduction of a large *Heracleum* species to northern Norway dating to the 1830′s, *H*. *persicum* has been a part of our northern flora for less than 180 years. It was popular as an ornamental during the second part of the 19th century, and brought to many stations along the coast. Sixty to seventy years after its introduction, it was well established outside gardens at least in Harstad and Tromsø – and has continued to expand ever since, now occurring at hundreds of localitites in Norway, mainly in Trøndelag (central Norway) and the three northernmost counties (Nordland, Troms and Finnmark).

Within less than two centuries, *H*. *persicum* has also become one of the few plants almost everyone in the north has heard of, gaining more than twenty different vernacular names, and a solid position in the childhood memories of many of those who have grown up here [75]. By doing so, it also clearly demonstrates that ethnobotany is not a discipline restricted to past or dying traditions. Given suitable material, e.g. a conspicuous alien, even modern town and city dwellers may coin, borrow, re-use or re-shape a variety of vernacular names and plant lore.

Disregarding local sentiments, *H*. *persicum* has been duly black-listed in Norway [16], being strongly invasive and an obvious threat to indigenous plants and vegetation types, which are rapidly transformed not least due to its allelopathic effect on surrounding plants [124-126], cf. also [127], and the heavy shading produced by the dense foliage. In combination with sunlight, the plant’s sap may cause severe burns [128-131]. Such sores frequently feature in newspaper notes, though *H*. *persicum* may seem less dangerous in this respect than *H*. *mantegazzianum*.

In terms of attempts of eradication, people need not worry. The stands of *H*. *persicum* are so large and widely distributed that eradication is inconceivable, and the cost alone would be prohibitive. Thus, new generations of children will certainly be able to use it in their games, probably adding more local lore in the process. Perhaps the species will even turn out to be useful (from an adult perspective) after all. Known as *golpar* in Iran, it is utilized there as a food plant. The fruits are commonly used as a spice, and young stems are harvested for making pickles [132]. In Iranian folk tradition, the plant is considered of medicinal importance, and recent studies have confirmed that it contains compounds of pharmacological interest [132-134].

## Competing interests

The author declares that he has no competing interests.

## Supplementary Material

Additional file 1 Decoding EBATA, or some notes on my own material. Click here for file

## References

[B1] Signorini M Piredda M Bruschi P Plants and traditional knowledge: An ethnobotanical investigations on Monte Ortobene (Nuoro, Sardinia) J Ethnobiol Ethnomed 2009 5 6 10.1186/1746-4269-5-6 19208227 PMC2661884

[B2] Singh AG Kumar A Tewari DD An ethnobotanical study of medicinal plants used in Terai forest of western Nepal J Ethnobiol Ethnomed 2012 8 19 10.1186/1746-4269-8-19 22591592 PMC3473258

[B3] Tabuti JRS Kukunda CB Kaweesi D Kasilo OMJ Herbal medicine use in the districts of Nakapiripirit, Pallisa, Kanungu, and Mukono in Uganada J Ethnobiol Ethnomed 2012 8 35 10.1186/1746-4269-8-35 22943789 PMC3484030

[B4] Bussmann RW Swartzinsky P Worede A Evangelista P Plant use in Odo-Bulu and Demaro, Bale region, Ethiopia J Ethnobiol Ethnomed 2011 7 28 10.1186/1746-4269-7-28 21943288 PMC3196683

[B5] Uprety Y Asselin H Dhakal A Julien N Traditional use of medicinal plants in the boreal forests of Canada: review and perspectives J Ethnobiol Ethnomed 2012 8 7 10.1186/1746-4269-8-7 22289509 PMC3316145

[B6] Belayneh A Asfaw Z Demissew S Bussa NF Medicinal plants potential and use by pastoral and agro-pastoral communities in Erer Valley of Babile Wereda, Eastern Ethiopia J Ethnobiol Ethnomed 2012 8 42 10.1186/1746-4269-8-42 23082858 PMC3549935

[B7] Svanberg I The use of rush (*Juncus*) and cotton-grass (*Eriophorum*) as wicks. An ethnobotanical background to a Faroese riddle Svenska landsmål och svenskt folkliv 1997 1998 145 15723596854

[B8] Pieroni A Pardo-de-Santayana M Svanberg I Luczaj L Anderson EN, Pearsall D, Hunn E, Turner N, Hoboken NJ History and current trends of ethnobiological research in Europe Ethnobiology 2011 New Jersey: Wiley-Blackwell 189 212

[B9] Vickery R Garlands, conkers and mother-die. British and Irish plant-lore 2010 London: Continuum Publishing

[B10] Łuczaj Ł Pieroni A Tardío J Pardo-de-Santayana M Sõukand R Svanberg I Kalle R Wild food plant use in 21st century Europe: the disappearance of old traditions and the search for new cuisines involving wild edibles Acta Soc Bot Pol 2012 81 359 370 10.5586/asbp.2012.031

[B11] Alm T Ethnobotany of *Linnaea borealis* (Linnaeaceae) in Norway Bot J Linn Soc 2006 151 3 437 452 10.1111/j.1095-8339.2006.00516.x

[B12] Alm T Iversen M Pardo-de-Santayana M, Pieroni A, Puri R Norway’s rosmarin (*Rhododendron tomentosum*) in past and present tradition Ethnobotany in the new Europe. People, health and wild plant resources 2010 New York Oxford: Berghahn Press 263 281

[B13] Pieroni A Quave C Nebel S Heinrich M Ethnopharmacy of the ethnic Albanians (Arbëreshë) of northern Basilicata, Italy Fitoterapi 2002 73 217 241 10.1016/S0367-326X(02)00063-1 12048017

[B14] Pieroni A, Vandebroek I Travelling cultures and plants: the ethnobiology and ethnopharmacy of migrations 2007 Oxford: Berghahn

[B15] Alm T Jensen C Often A Tromsøpalmens historie Ottar 2006 261 3 9

[B16] Gedderaas L Moen TL Skjelseth S Larsen LK Fremmede arter i Norge med norsk svarteliste 2012 2012 Trondheim: Artsdatabanken

[B17] Mabberley DJ Mabberley’s plant-book. A portable dictionary of plants, their classification and uses 2008 Cambridge: Cambrigde University Press

[B18] Wyse Jackson M Observations on the Irish distribution of a plant with serious public health implications: Giant hogweed (*Heracleum mantegazzianum* Sommier and Levier) Bull Ir Biogeogr Soc 1989 12 94 113

[B19] Clement EC Foster MC Alien plants of the British Isles 1994 London: Botanical Society of the British Isles

[B20] Jahodova Š Trybush S Pyšek P Wade M Karp A Invasive species of *Heracleum* in Europe: an insight into genetic relationships and invasion history Diversity Distrib 2007 13 99 114 10.1111/j.1366-9516.2006.00305.x

[B21] Pyšek P De Waal LC, Child LE, Wade PM, Brock JH Ecological aspects of invasion by *Heracleum mantegazzianum* in the Czech Republic Ecology and management of invasive riverside plants London: John Wiley & Sons

[B22] Andersen UV Healey MG, Doody JP Invasive aliens: a threat to the Danish coastal vegetation? Directions in European coastal managment 1995 Cardigan: Samara Publishing 335 344

[B23] Andersen UV 1995 Pyšek P, Prach K, Rejmánek M, Wade M Comparison of dispersal strategies of alien and native species in the Danish flora Plant invasions - general aspects and special problems 1995 Amsterdam: SPB Academic Publishing 61 70

[B24] Tiley GED Dodd FS Wade PM *Heracleum mantegazzianum* Sommier & Levier. (Biological flora of the British Isles No. 190) J Ecol 1996 84 297 319 10.2307/2261365

[B25] Andersen UV De Waal LC, Child LE, Wade PM, Brock JH Sheep grazing as a method of controlling *Heracleum mantegazzianum* Ecology and management of invasive riverside plants 1994 London: John Wiley & Sons 77 91

[B26] Andersen UV Calov B Long-term effects of sheep grazing on giant hogweed (*Heracleum mantegazzianum*) Hydrobiologia 1996 340 277 284 10.1007/BF00012768

[B27] Caffrey JM De Waal LC, Child LE, Wade PM, Brock JH Spread and management of *Heracleum mantegazzianum* (Giant Hogweed) along Irish river corridors Ecology and management of invasive riverside plants 1994 London: John Wiley & Sons 67 76

[B28] Calov B Andersen UV Bekæmpelse af Kæmpe-Bjørneklo Miljø- og Ener-giministeriet, Forskningscentret for skov & landskab, videnblad 1996 6 0 416821046

[B29] Tiley GED Wade PM De Waal LC Dodd FS De Waal LC, Child LE, Wade PM, Brock JH Control and management of Giant Hogweed (*Heracleum mantegazzianum*) Ecology and management of invasive riverside plants 1994 London: John Wiley & Sons 111 126

[B30] Sampson C De Waal LC, Child LE, Wade PM, Brock JH Cost and impact of current control methods used against *Heracleum mantegazzianum* (Giant Hogweed) and the case for investigating a biological control programme Ecology and management of invasive riverside plants 1994 London: John Wiley & Sons 55 65

[B31] Tiley GED Philp B De Waal LC, Child LE, Wade PM, Brock JH *Heracleum mantegazzianum* (Giant Hogweed) and its control in Scotland Ecology and management of invasive riverside plants 1994 London: John Wiley & Sons 101 109

[B32] Williamson JA Forbes JC Giant Hogweed (*Heracleum mantegazzianum*). Its spread and control with glyphosat in amenity areas Proceedings British crop protection conference – Weeds 1982 967 972

[B33] Elven R Alm T Berg T Båtvik JII Fremstad E Pedersen O Johannes Lid & Dagny Tande Lid: Norsk flora 2005 7 Oslo: Det norske samlaget

[B34] Fremstad E Elven R De store bjørnekjeksartene *Heracleum* i Norge NTNU Vitenskapmuseet, Rapp Bot Ser 2006 2006 2 1 35

[B35] Fröberg L Jonsell B, Karlsson T *Heracleum* L Flora Nordica. Vol. 6. Thymelaeaceae to Apiaceae 2010 Stockholm: The Swedish Museum of Natural History 224 234

[B36] Alm T Often A Tromsøpalmen og dens slektninger – et knippe pestplanter Ottar 2006 261 10 17

[B37] Engelskjøn T Skifte O The vascular plants of Troms, North Norway. Revised distribution maps and altitude limits after Benum: The flora of Troms fylke Tromura Naturvitensk 1995 80 1 227

[B38] Alm T Jensen C Tromsøpalmen (*Heracleum laciniatum*) – noen kommentarer til artens innkomst og ekspansjon i Nord-Norge Blyttia 1993 51 61 69

[B39] Often A Graff G Skillekarakterer for kjempebjørnekjeks Heracleum mantegazzianum og tromsøpalme H. laciniatum Blyttia 1994 52 129 133

[B40] Øvstedal, DO Er tromsøpalma sit namn *Heracleum persicum* Desf.? Polarflokken 1987 11 1 25 26

[B41] Øvstedal DO Om fruktkarakterar hos tromsøpalme og kjempebjønnkjeks Polarflokken 1997 21 2 167 170

[B42] Øvstedal DO Tre slag bjønnkjeks (*Heracleum*) i Tromsø Polarflokken 1985 9 83 89

[B43] Alm T Kulturspredte arter i Harstad og Bjarkøy (Troms) – en kartlegging i 2007–2010. 2. Artsomtaler, ertefamilien (Fabaceae) til kaprifolfamilien (Caprifoliaceae) Polarflokken 2010 32 2 49 91

[B44] Alm T Gamst SB Gamst URB Sortland A Kulturspredte arter i Tromsø ved starten av et nytt årtusen. 1. Innledning og artsomtaler: Hampefamilien (Cannabaceae) til skjermplantefamilien (Apiaceae) Polarflokken 2004 28 1 3 98

[B45] Often A Kjempebjørnekjeks (*Heracleum mantegazzianum*) funnet i Tromsø Polarflokken 1994 18 49 53

[B46] Engelskjøn T Kontroll av en aggressiv nykomling i Tromsø, *Heracleum mantegazzianum* Polarflokken 1995 19 100 102

[B47] Christy W Notes of a voyage to Alten, Hammerfest, &c Entomol Mag 1837 4 462 483

[B48] Nelson EC Small ad for giant hogweed B.S.B.I. News 1991 57 26 27

[B49] Fries TM En botanisk resa i Finmarken 1864 Bot Not 1865 1865 1 6 16 (2):27–38,(3):42–58

[B50] Von Heuglin MT Reise in Norwegen und Spitzbergen im Jahre 1870 1872 Braunschweig: Druck und Verlag von George Westermann

[B51] Escard F Le prince Roland Bonaparte en Laponie. Episodes et tableaux 1886 Paris: Imprimé pour l’auteur par G. Chameroy

[B52] Sewell P The Flora of the Coasts of Lapland and of the Yugor Strait (N.-W. Siberia) as observed during the Voyage of the “Labrador” in 1888, with Summarised List of all the Species known from the Islands of Novaya Zemlya and Waigatz, and from the North Coast of Western Siberia Transact Proceed Bot Soc Edinburgh 1889 17 444 481 10.1080/03746608909468374

[B53] Schübeler FC Die Pflanzenwelt Norwegens. Ein Beitrag Zur Natur- und Culturgeschichte Nord-Europas 1873-1875 Christiana (Oslo): Universitets-Program

[B54] Schübeler FC Viridarium Norvegicum. Norges væxtrige. Et bidrag til Nord-Europas natur- og culturhistorie. Vol. 2 1888 Christiania (Oslo): Universitetsprogram

[B55] Anonymous Vår på Sørøya 1894 Øyfolk 2006 2006 88 89

[B56] Reusch H Folk og natur i Finmarken 1895 Kristiania: T.O. Brøgger

[B57] Helland A Norges land og folk. Topografisk-statistisk beskrivelse over Finmarkens amt. Vol. 1 1905 Aschehoug: Kristiania (Oslo)

[B58] Østerud T Kjemisk undersøkelse av planten *Heracleum panaces* foretatt ved Forsøksgården Holt Meld Statens Forsøksgard Holt 1934 1935 59 70

[B59] Nilsen EW Tromsøpalmen Polarflokken 1991 15 149 150

[B60] L Tromsø i 60-årene Folkevennen 1899 47 7 337 356

[B61] Notø A Florula Tromsøensis Editio nova. Tromsø Mus Aarsh 1901 15 157 174

[B62] Alm T Trollbær og tortengress. Folkeminne fra Klatran og Sørvikmark. Om planter og plantenavn Håløygminne 1983 16 373 396

[B63] Lynge B Om vaarens fremadskriden i Finmarken i juni 1914 Nyt Mag Naturvidensk 1915 52 357 379

[B64] Nordhagen R Norsk flora 1940 Oslo: Aschehoug

[B65] Lid J Norsk flora 1944 Oslo: Det norske samlaget

[B66] Lid J Norsk flora 1952 2 Oslo: Det norske samlaget

[B67] Lid J Norsk og svensk flora 1963 3 Oslo: Det norske samlaget

[B68] Avrorin NG Andrejev GN Golovkin BN Kalnin AA Introduction of plants to the polar north, part I 1964 Kirovsk: Kola science center/Polar-alpine botanical garden [In Russian]

[B69] Grenand D Le voyage des mots. Logique de la nomination des plantes: examples dans les tupi du Brésil Rev Ethnoling 1995 7 23 28

[B70] Van den Eynden V Cueva E Cabrera O Of “climbing peanuts” and “dog’s testicles”, mestizo and Shuar plant nomenclature in Ecuador J Ethnobiol 2004 24 279 306

[B71] Høeg OA Planter og tradisjon. Floraen i levende tale og tradisjon i Norge 1925–1973. Oslo - Bergen –1974 Tromsø: Universitetsforlaget

[B72] Christiansen H Det norrøne ord lúðr Maal og minne 1952 1952 101 106

[B73] Olberg G Pflanzen und Tiere in Norwegen. 1. Teil – Das Pflanzenleben Norwegen 1944 Oslo: Deutsche Zeitung in Norwegen

[B74] Christiansen H Ordene lur og stokk i moderne norsk Maal og minne 1952 1952 107 122

[B75] Alm T Tromsøpalmen i folketradisjonen Ottar 2006 261 18 24

[B76] Grødahl S Rydd opp i Saraholla! Tromsø 2006 108 120 3623800181

[B77] Anonymous Sommer paa den 70de Breddegrad Morgenbladet 1900 1900 28 110 Extranumer

[B78] Pollestad E Skulptøren som oppdaget tromsøpalmen Nordlys 1994 93 153 16

[B79] Pollestad E Mangfoldets «Nordnorsken Nordlys 1995 94 42 14

[B80] Pollestad E Natur ved vegtunnel Nordlys 1998 97 159 14

[B81] Pedersen G Du klarer ikke å gjette hva denne skulpturen er laget av Nordlys 2012 111 138 47

[B82] Hestvik L Prydplante og miljøproblem Brønnøysund avis 2011 9123802222

[B83] Johansen R author Barndom og ungdom i krig og fred 2007 Tromsdalen

[B84] Wessel-Hansen S Stor fest for «Storskogrampen Nordlys 2006 105 49 16 17

[B85] Hochlin J Artig etterpå. Muntre epistler og vers til trøst i motbakken 2006 Tromsøboka: Tromsø

[B86] Jonsson H Bevaringsverdige tromsøpalmen Tromsø 2007 109 166 323800181

[B87] Hochlin J Vær ikke bekymret Nordlys 2007 106 214 44

[B88] Łuczaj JŁ Kujawska M Botanists and their childhood memories: an underutilized expert source in ethnobotanical research Bot J Linn Soc 2012 168 334 343 10.1111/j.1095-8339.2011.01205.x

[B89] Hochlin J Aroma Nostalgia Nordlys 2001 100 294 18

[B90] Mørkved B Hanssen Ø Lyd og ulyd av planter Ottar 2007 266 44 47

[B91] Enoksen R Riddu Riddu er i gang Nordlys 2002 101 154 28 29

[B92] Bruun O Lager instrument av forhatt plante Harstad tidende 2005 119 135 52 53

[B93] Enoksen R Balsfjord en hvit flekk Nordlys 2007 106 123 51

[B94] Olsen LR Strupe av sølv Altaposten 2009 41 81 14

[B95] Bruun O Kulturkveld i Sandtorg kirke Harstad tidende 2010 123 163 25

[B96] Anonymous Slik blir du kvitt palmen Tromsø 2007 109 159 923800181

[B97] Sandnes K Mener palmene må tas tidligere Tromsø 2007 109 160 523800181

[B98] Roksøy LL Slik kan du bekjempe palmen Harstad tidende 2007 121 171 5

[B99] Simonsen MH Til kamp mot Tromsøpalmen Troms folkeblad Sommermagasinet 2009 45 34 35

[B100] Hedlund IK Botemiddel mot tromsøpalmen Nordlys 2005 104 174 32

[B101] Hårek Plagsom tromsøpalme Nordlys 2008 107 115 41

[B102] I.S Tromsøpalmen Tromsø 2009 111 169 223800181

[B103] Ken Tromsøpalmen Tromsø 2008 110 223 223800181

[B104] Siri L Tromsøpalme Tromsø 2007 109 161 323800181

[B105] Hansen C Tar tromsøpalmen i forsvar Tromsø 2009 111 167 623800181

[B106] Hanssen HI Rydder i palmeskogen Nordlys 2006 105 184 4

[B107] Graff G Litt om Tromsøpalmen Tromsø 2007 109 167 32 3323800181

[B108] Fjellrein Latterlig Tromsø 2008 110 223 167 223800181

[B109] LL Tromsøpalmen Tromsø 2009 111 167 223800181

[B110] Graff G Gjør tromsøpalmen til en skattet parkplante Tromsø 2010 112 174 4723800181

[B111] Moe I Frodig og fritt Nordlys 1997 96 169 17

[B112] Sandnes K Mye farligere enn tromsøpalmen Tromsø 2007 109 166 8 923800181

[B113] K.S Palmens venner Tromsø 2009 111 166 323800181

[B114] Hagerup B Palmene ikke farlige Tromsø 2007 109 169 323800181

[B115] Eilertsen WH Tromsø-palmen Tromsø 1998 101 108 3623800181

[B116] Wilhelmsen B Tromsøpalmen i byvåpenet? Nordlys 2002 101 136 20

[B117] Wilhelmsen B Byvåpen Tromsø 2005 108 173 323800181

[B118] L.M.L Byvåpen Tromsø 2009 111 169 223800181

[B119] F.W Byvåpen Tromsø 2009 111 170 323800181

[B120] L.L Vrak reinen Tromsø 2009 111 170 323800181

[B121] H.K Bruk brua Tromsø 2009 111 170 323800181

[B122] Wilhelmsen B Byvåpenet Tromsø 2011 113 13 4523800181

[B123] Barth-Heyerdahl Ø, Johnsen O Gi kronprisparet ei tromsøpalme Nordlys 2001 101 171 10 11

[B124] Junttila O Allelopathy in *Heracleum laciniatum*: Inhibition of lettuce seeed germination and root growth Physiol Plant 1975 33 22 27 10.1111/j.1399-3054.1975.tb03758.x

[B125] Junttila O Allelopathic inhibitors in seeds of *Heracleum laciniatum* Physiol Plant 1976 36 374 378 10.1111/j.1399-3054.1976.tb02259.x

[B126] Myrås H Junttila O Interaction between *Heracleum laciniatum* and some other plants Holarctic Ecol 1981 4 43 48

[B127] Bærheim-Svendsen A Blyberg M Cumarine der Wurzeln von *Heracleum panaces* Pharm Acta Helv 1959 1959 33 36 13633431

[B128] Kavli G Photoreactivity of Heracleum laciniatum. An experimental study evaluating skin reactions and in vitro phototoxicity of plant material and isolated furocoumarins PhD Thesis, University of Troms 1982

[B129] Kavli G Volden G Phytophotodermatitis. Photodermatology 1984 1984 65 75 6397734

[B130] Anonymous Forbrent av Tromsøpalme Dagbladet 2008 140 174 17

[B131] Arnesen IA Farlig tromsøpalme Tromsø 2007 109 168 323800181

[B132] Hemati A Azarnia M Angaji SA Medicinal effects of *Heracleum persicum* (Golpar) Middle-East J Sci Res 2010 5 17 176

[B133] Sayyah M Moaied S Kamalinejad M Anticonvulsant activity of *Heracleum persicum* seed J Ethnopharmacol 2005 98 209 11 10.1016/j.jep.2004.12.026 15763386

[B134] Sharififar F Pournourmohammadi S Arabnejad M Rastefarianzadeh R Ranjbaran O Purgemmaty A Immunomodulatory activity of aqueous extract of *Heracleum persicum* Desf. in mice Iran J Pharm Res 2009 8 287 292

