# Supplementary material for: Ethnobotany of Heracleum persicum Desf. ex Fisch., an invasive species in Norway, or how plant names, uses, and other traditions evolve
Source: J Ethnobiol Ethnomed. 2013 Jun 24;9:42. doi: 10.1186/1746-4269-9-42 (PMC3699400; doi:10.1186/1746-4269-9-42)
Supplement: Additional file 1 — Decoding EBATA, or some notes on my own material. [file 1746-4269-9-42-S1.docx]

The material found in my own collections (EBATA = Ethnobiological Archive T. Alm) is as diverse as the ethnobiological traditions it reflects. I was brought up in a family where, as is (or was) common in Norway, visiting the outdoors, collecting berries, fishing, and so forth, was an integral part of life. This stimulated an early interest in nature. In terms of ethnobotany, I started collecting data in 1977, at first mainly from older members of my own family. This work was at least partly inspired by the 1974 publication of Ove Arbo Høeg’s *Planter og tradisjon* (‘Plants and tradition’), containing his vast personal collection of Norwegian plant names and other ethnobotanical data. If I remember correctly, I got a copy of this book as a birthday present from my grandma – and little did she know the number of questions she and others would be burdened with.

Contrary to Høeg, I have not restricted my interest to plants (and fungi). I also at once embarked on collecting traditions related to animals – and so I have done ever since. For that matter, asking about animals may yield information on plants that would otherwise remain unrecorded. If I had not asked people at Arnøya in northernmost Troms about the basking shark *Cetorhinus maximus* Gunn.*,* I would not have known that they put bundles of *Angelica archangelica* L. in their boats when going to sea. This vast shark was (wrongly) perceived as dangerous, but supposedly fled when the bundles were thrown into the sea.

Due to my work as curator of vascular plants at Tromsø museum, University of Tromsø, I have travelled extensively in the northern parts of Norway, in particular in the counties of Troms and Finnmark. The latter county, Norway’s largest (and larger than e.g. Denmark or Switzerland), had almost no coverage in the collections of O.A. Høeg. His study also largely neglected the existence of ethnic minorities in Norway, and both the Sámi and Finnish minorities certainly possess extensive ethnobotanical traditions.

I have tried to fill the gaps left by Høeg, both in terms of geography, ethnicity and languages, though I certainly have not refrained from collecting whatever useful material I could locate, no matter its origin, from the far south of Norway to country’s sparsely populated north. Some notes contain snippets of information gathered during travels, conversations and so forth, but the real bulk of my material derives from an effort at gathering extensive data sets from individual informants and localities.

For this purpose, I have used two self-made, illustrated questionnaires (in several versions). Both list a selection of species and species groups likely to occur in folk tradition, with some additional questions on more general topics. The first questionnaire includes about 150 plants and fungi, and the second a little more than 200 animal taxa. These questionnaries have also been used as a guide to memory during all the more extensive interviews. Getting through each of them may take anything from two or three hours to two or three days.

Some of my informants derive from casual encounters during general botanical field work – and other travels. Quite a few have been recruited by requesting supplementary information from people who have written or commented on specific topics, e.g. in newspapers, journals etc. Others have been selected according to local advice, especially during field work devoted to ethnobiology.

The resulting material may show some overrepresentation of old females – not necessarily because they know more than men or younger people, but simply because they generally live longer, and thus are the oldest (and sometimes only) individuals in smaller settlements. To my experience, gender means less in terms of knowledge than interest. Some individuals are more inquisitive than others, and tend to accumulate knowledge and beliefs, including those related to plants, fungi, and animals. Others have a more restricted area of interest, as so clearly put by an 88 year old Norwegian fisherman from Ekkerøya in Vadsø, Finnmark (and despite the fact that he knew a fair selection of plant names and traditions): he was only interested in what was found from sea-level down.

It is generally difficult to persuade people into writing answers. Like Høeg, I have found that old school-teachers provide the only consistent exception to this rule. On the other hand, I have had little or no difficulty in getting people to answer the same questions orally, during more or less formal interviews. These also have the obvious advantage that promising fields or tracks can be pursued at once, and dead ends abandoned. In addition, ill-defined ethnobiological entities may be clarified by tracking down the species or species group in question in the vicinity, adding voucher specimens in the process.

To give some impression of the resulting material, I will use the records from 2006 (EBATA 2006:1-146), which contain numerous notes on *Heracleum,* as an example Of the 146 records included, 23 are letters or e-mail notes, five letters accompanied by filled-out questionnaires, 27 are interviews, often extensive, five are short interviews on specific topics carried out in the course of telephone conversations, and the remaining 86 are brief notes on one or a few species or topics, made e.g. during casual conversations. In terms of geographical distribution, the 2006 material is predominated by data from northern Norway, but also comprise substantial data sets from SE Norway (Østlandet, ten records) and W Norway (Vestlandet, ten records, partly very extensive), with some additional snippets from southernmost Norway (Sørlandet, a single list of plant and animal names) and central Norway (Trøndelag, a single note). Most records reflect ethnic Norwegian traditions, but the material also includes substantial data on Finnish (seven records) and Sámi tradition (21 records). The vast majority of the information pertains to ethnobotany, although some twenty records also (or only) contain ethnozoological data, including some extensive contributions.

The entire collection is currently stored in the vaults of Tromsø museum; much of it also in various digital formats. Only minor parts have been published (and hardly any of the ethnozoological records). The material is not likely to become generally available until I have finished working with it, and is thus now only available at my discretion.
